# Supplementary material for: Deletion of wfs1 Impairs Oligodendrocyte Precursor Cells Dorsal Distribution and Myelination Through the wfs1-hmgcs1 Axis in Zebrafish
Source: Biology (Basel). 2026 Aug 16;15(16):1402. doi: 10.3390/biology15161402 (PMC13510151; doi:10.3390/biology15161402)
Supplement: Supplementary file 1 [file biology-15-01402-s001.zip › File S1-Transcriptome sequencing data and differentially expressed genes (DEGs) in wfs1-- zebrafish at 5 dpf/2.KEGG/wfs1KOvsWT/DOWN/src/dre04068.html]

dre04068


- dre:100000252

- Down regulated genes

100000252\_1(-1.7718817168434)

- dre:571431

- Down regulated genes

571431(-6.42862549702529)

- dre:563708

- Down regulated genes

563708(-3.59233336704936)

- dre:245702

- Down regulated genes

245702\_1(-1.45433245048201)

- dre:431763

- Down regulated genes

431763\_1(-4.20440659354098)

- dre:431763

- Down regulated genes

431763\_1(-4.20440659354098)

- dre:100006858

- Down regulated genes

100006858\_1(-1.49071226984431)

- dre:100000242

- Down regulated genes

100000242(-6.34512572021214)

- dre:324140

- Down regulated genes

324140(-5.90052475401947)

Close
